# NCG 5.0: updates of a manually curated repository of cancer genes and associated properties from cancer mutational screenings

**DOI:** 10.1093/nar/gkv1123

**Published:** 2015-10-29

**Authors:** Omer An, Giovanni M. Dall'Olio, Thanos P. Mourikis, Francesca D. Ciccarelli

**Affiliations:** Division of Cancer Studies, King's College London, London SE11UL, UK

## Abstract

The Network of Cancer Genes (NCG, http://ncg.kcl.ac.uk/) is a manually curated repository of cancer genes derived from the scientific literature. Due to the increasing amount of cancer genomic data, we have introduced a more robust procedure to extract cancer genes from published cancer mutational screenings and two curators independently reviewed each publication. NCG release 5.0 (August 2015) collects 1571 cancer genes from 175 published studies that describe 188 mutational screenings of 13 315 cancer samples from 49 cancer types and 24 primary sites. In addition to collecting cancer genes, NCG also provides information on the experimental validation that supports the role of these genes in cancer and annotates their properties (duplicability, evolutionary origin, expression profile, function and interactions with proteins and miRNAs).

## INTRODUCTION

Cancer genome projects, including The Cancer Genome Atlas (TCGA, https://tcga-data.nci.nih.gov/) and the International Cancer Genome Project (ICGC, https://dcc.icgc.org/) have so far mapped DNA alterations in more than 13 000 cancer samples. These massive sequencing efforts show that somatic modifications vary greatly between and within cancer types (1–3). Only some of the acquired alterations, however, confer a selective advantage that promotes cancer development (*driver alterations*). The large majority of alterations have no or little role in cancer and are fixed in the cancer genome as a by-product of the selection acting on drivers (*passenger alterations*). One of the challenges of cancer genomics is to effectively distinguish between driver and passenger alterations in order to identify the molecular determinants of cancer. Most known driver alterations modify protein-coding genes (*cancer genes*). The ability to identify cancer genes among the wealth of mutated genes is crucial to better understand cancer biology and to empower the development of innovative anti-cancer therapy.

Network of Cancer Genes (NCG) is a database launched in 2010 with the aim to collect cancer genes from the literature. Curators constantly review cancer mutational screenings and annotate altered genes that either have well-established cancer functions (*known cancer genes*) or are putative cancer drivers (*candidate cancer genes*). Originally (4), NCG collected data from only five mutational screenings and annotated most known cancer genes from the Cancer Gene Census (CGC) (5). The last five years have seen the rapid accumulation of cancer genomic data from thousands of samples, with almost all human genes mutated in at least one sample (6,7). Due to this overwhelming amount of data and to avoid the inclusion of mutated genes with no role in cancer, in this release we have substantially reviewed the procedure to identify cancer genes. NCG now collects 1571 cancer genes, 518 of which are known cancer genes. The remaining 1053 genes are candidate cancer genes whose driver role has been predicted in the original publication using a variety of methods (Supplementary Table S1). Given the importance of a robust experimental support for the cancer activity of candidate cancer genes, NCG now collects additional literature describing available orthogonal validations. NCG also annotates various properties of cancer genes such as the presence of extra copies in the genome (gene duplicability), the evolutionary origin, the connectivity of the encoded proteins in the protein–protein and miRNA interaction networks, and the comprehensive gene expression profile across 38 human tissues and 1543 cancer cell lines.

The manual curation of the literature to extract cancer driver genes and the annotation of a large number of additional properties make NCG a comprehensive and updated resource to navigate the overwhelming amount of cancer data with a particular focus on the genetic determinants of cancer.

## MANUAL ANNOTATION OF CANCER GENES

**Figure 1.**
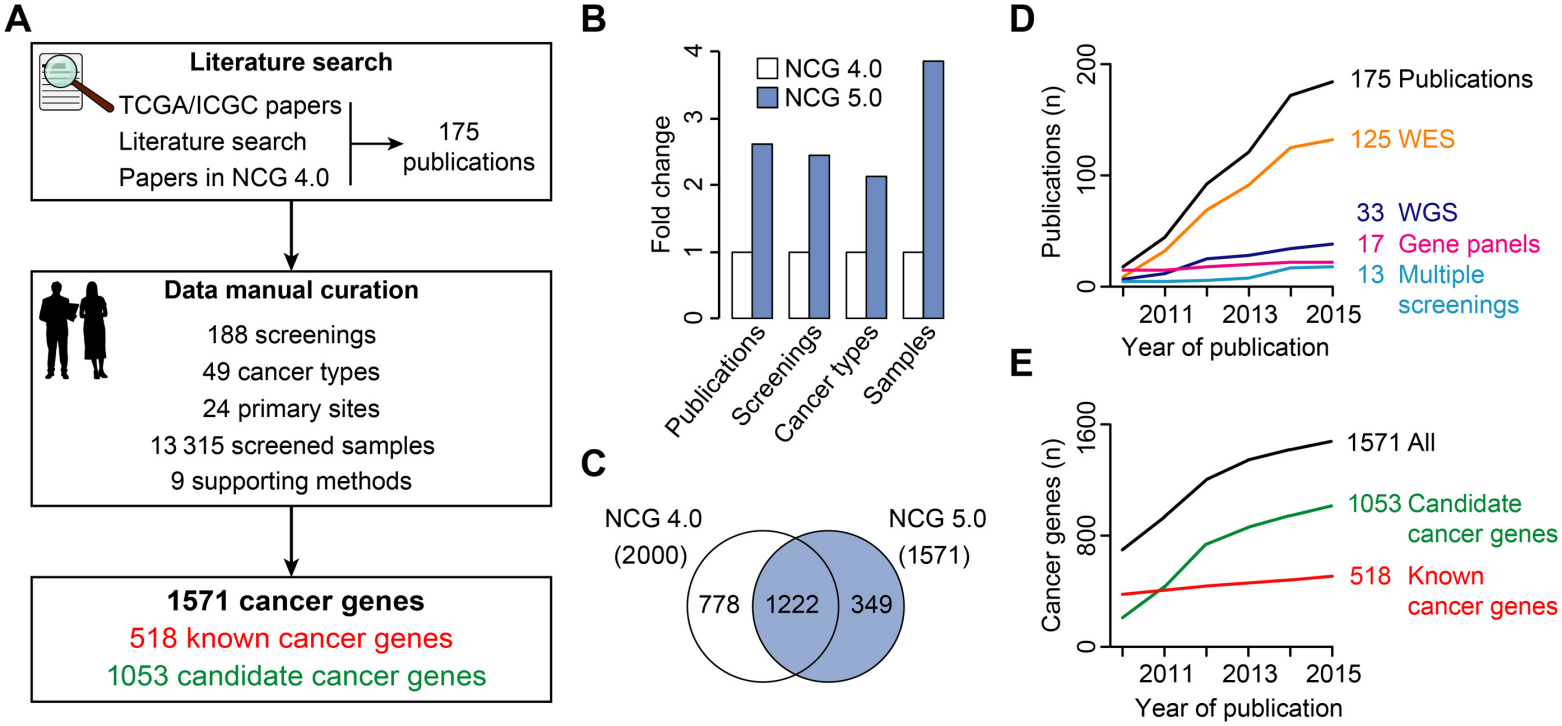
Curation procedure and comparison between NCG 5.0 and NCG 4.0: (**A**) Flowchart of the curation procedure used in NCG. After the identification of relevant publications describing cancer mutational screenings, two independent curators extract cancer genes and related information on types of screening and cancer, primary sites, screened samples and supporting methods. (**B**) Number of publications, screenings, cancer types and screened samples in NCG 5.0 as compared to NCG 4.0. (**C**) Venn diagram of cancer genes in NCG 4.0 and NCG 5.0. The reasons for the removal of 778 genes from the database are detailed in Supplementary Table S2. (**D–E**) Growth of NCG data in time. Shown are the number of publications, screenings and cancer genes starting from 2010, year of the first release of NCG. All screenings that were published prior of 2010, were collapsed.

In this release of NCG, the procedure for the inclusion of cancer genes in NCG has been reviewed and standardized (Figure 1A). The first difference with previous versions is to restrict the inclusion only to studies that describe mutational screenings of cancer samples and that distinguish between cancer genes and genes with passenger mutations. This led to the identification of 119 new publications. To be consistent with these inclusion criteria, all 68 studies present in the previous release were re-analysed. Twelve of them were excluded because they screened cancer cell lines rather than cancer samples or used no methods to identify cancer genes among all mutated genes. As a result of this extensive literature search, NCG 5.0 currently collects 175 studies (Supplementary Table S1). Two curators reviewed independently each publication to extract cancer genes and complementary information, such as the screening and the cancer types, the primary sites, the number of sequenced samples and the methods that were applied to identify cancer genes (Figure 1A). This manual curation resulted in 1260 cancer genes, 207 of which were annotated as known cancer genes in CGC. The remaining 1053 genes were candidate cancer genes identified in the original study using one or more methods (Supplementary Table S1). Additional known cancer genes were also added from CGC (February 2014), leading to a total of 1571 cancer genes. If information was available, cancer genes were further annotated as dominant (mostly oncogenes) or recessive (mostly tumour-suppressors) genes.

As compared to NCG 4.0 (8), NCG 5.0 now collects information from more than the double number of publications, screenings and cancer types and from four times more cancer samples (Figure 1B). Despite this substantial increase of data, the number of cancer genes decreased from 2000 to 1571 (Figure 1C), because of the more restrictive criteria. In particular, 612 genes were removed because the original publication was excluded and 166 genes because they had no support as cancer drivers (Supplementary Table S2). Overall, the studies in NCG 5.0 describe 188 mutational screenings, including 125 whole exome sequencings, 33 whole genome sequencings, 17 screenings of selected gene panels and 13 screenings based on multiple approaches (Figure 1D). Interestingly, the number of cancer genes with a well-documented role in cancer increases at a much slower pace as compared to candidate cancer genes (Figure 1E). This highlights the currently unmet need of efficient experimental assays that support the predicted role of candidate genes in cancer.

**Figure 2.**
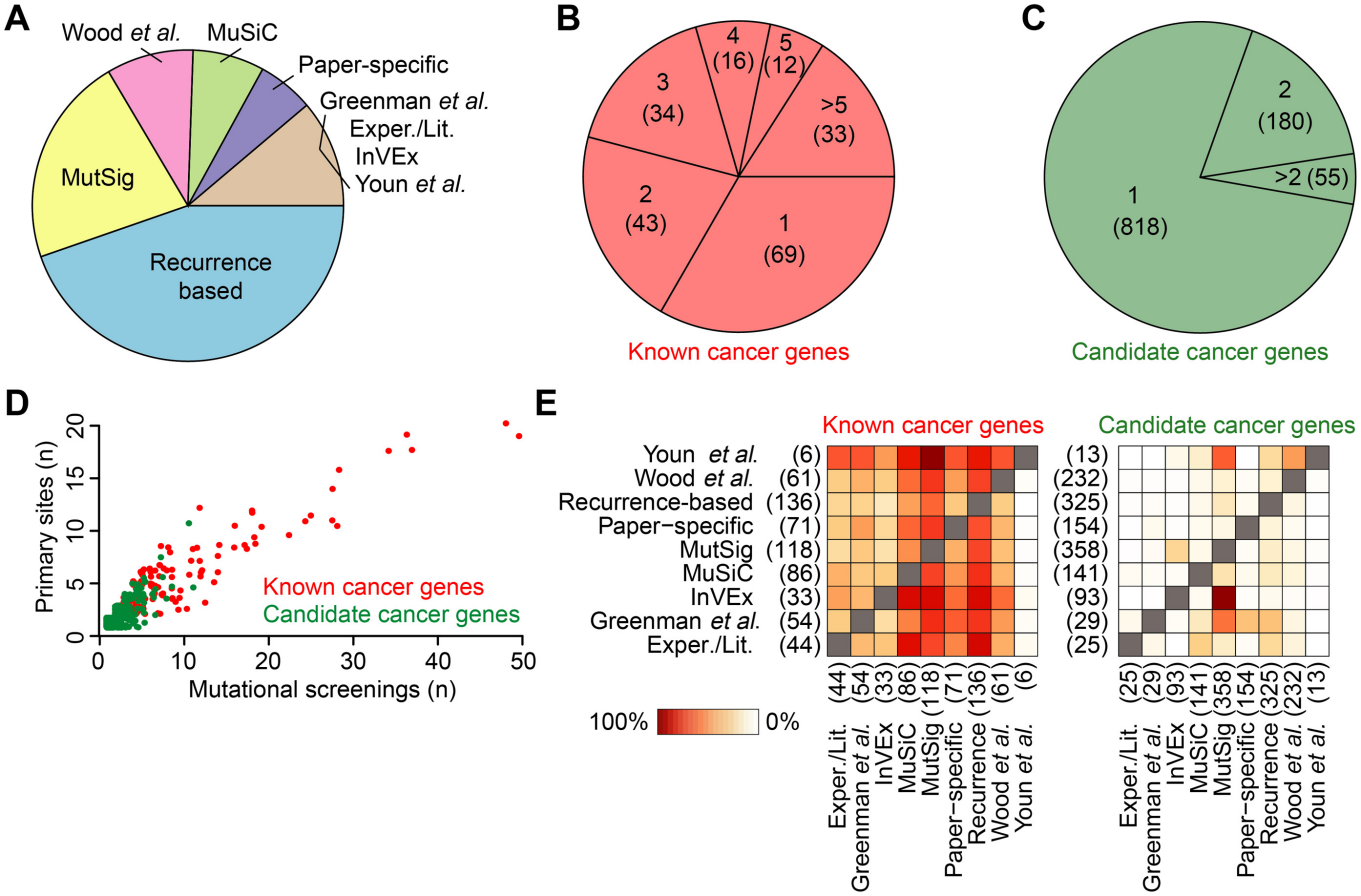
Overview of data in NCG 5.0: (**A**) Cancer mutational screenings divided according to the method that was applied to identify cancer genes in the original publication. Methods and corresponding screenings are described in Supplementary Table S1. (**B–C**) Fractions of known and candidate cancer genes supported by one or more methods. Gene counts are reported in brackets. (**D**) Number of mutational screenings and primary sites where each cancer gene has been reported as a driver. *TP53* is an outlier and has been excluded from the analysis because it has been identified in 113 screenings across 22 primary sites. (**E**) Heatmaps of the overlap between methods identifying known and candidate cancer genes. Each box represents the percentage of cancer genes identified with one method that are also supported by another. For each method, the total number of associated cancer genes is reported in brackets.

Almost all mutational screenings collected in NCG 5.0 applied only one method to identify cancer genes (Supplementary Table S1). The most common was the recurrence of mutation of a given gene across samples, which was taken as a sign of functional selection (Figure 2A and Supplementary Table S1). Other commonly used methods included MutSig (6) and MuSiC (9) (Figure 2A and Supplementary Table S1). Interestingly, the majority of known cancer genes (67%) had the support of at least two methods (Figure 2B), while most candidate cancer genes (78%) have been predicted by only one method (Figure 2C). In agreement with this, known cancer genes were overall identified as drivers across a higher number of mutational screenings and primary cancer sites as compared to candidate cancer genes (Figure 2D). The tendency of candidate cancer genes to be cancer specific was also reflected by the lower overlap between methods that support them as compared to those that support known cancer genes (Figure 2E). Cases where the overlap was higher (i.e. between MutSig and Invex, Figure 2E) corresponded to screenings where both methods were used (Supplementary Table S1).

## EXPERIMENTAL VALIDATION OF CANDIDATE CANCER GENES

Candidate cancer genes that are identified using computational methods often lack additional experimental validation of their cancer driver role. The main reason is that functional follow-ups are often cumbersome and require *ad hoc* design for individual genes. The experimental proof of predicted driver role is however crucial for the translatability of potentially relevant discoveries into increased knowledge and novel treatments.

**Figure 3.**
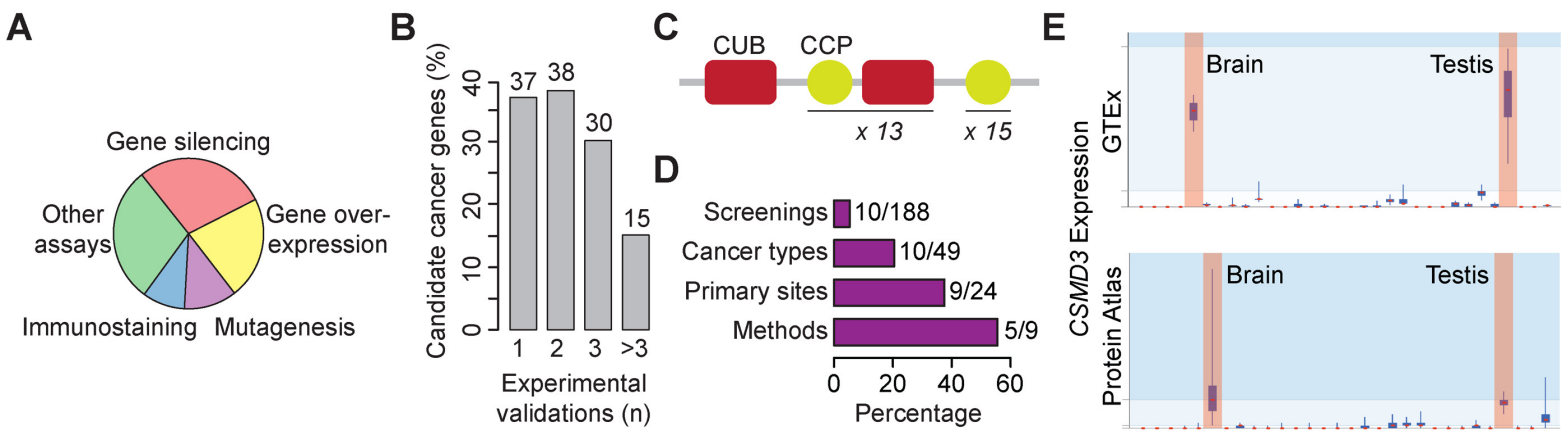
Validation of candidate cancer genes and alteration spectrum of *CSMD3*: (**A**) Fractions of validated candidate cancer genes according to the used experimental assay. Gene silencing refers to stable knockout or transient knockdown via RNA interference. Other assays include *in silico* protein modelling, survival analysis, drug response, protein activity, rhotekin pull-down and xenograft cancer models. (**B**) Percentage of candidate cancer genes that have been validated using one or more experimental approaches. The corresponding number of genes is shown above each bar. The full list of experiments and genes is reported in Supplementary Table S3. (**C**) Protein domain architecture of CSMD3 according to the SMART database (32). (**D**) Percentage of mutational screenings, cancer types, primary sites and methods that support the cancer driver role of *CSMD3*. Corresponding numbers are provided. (**E**) Expression profile of *CSMD3* in normal human tissues. Tissues where the gene is expressed in GTEx and Protein Atlas are highlighted in red.

In this release of NCG, we have extensively reviewed the literature to search for experimental validations of candidate cancer genes. NCG now annotates available orthogonal experiments that have been performed in the original study or in follow-up studies for 120 out of 1053 candidate cancer genes (11% of the total, Table 1 and Supplementary Table S3). Most commonly used approaches measure the effect of gene silencing or gene overexpression in cell lines (Figure 3A and Supplementary Table S3) and the majority of candidate genes (83 out of 120) have been validated through multiple assays (Figure 3B).

**Table 1.** Experimental validation of candidate cancer genes

Experimental validation	Candidate cancer genes (n)	Publications (n)
Gene overexpression	60	74
Transient RNA interference	58	52
Mutagenesis	31	41
Immunostaining	25	26
Stable gene knockout	23	22
Survival analysis	20	21
Protein activity assay	19	20
Drug response assay	15	17
*In silico* protein modelling	12	14
Xenograft	10	11
Rhotekin pull-down	2	5
Total	275 (120 unique genes)	303 (166 unique publications)

For each type of experimental validation, the numbers of validated candidate genes and corresponding publications are shown. The complete gene list with references to the original papers is given in Supplementary Table S3.

An interesting case is *CSMD3*, the gene associated with benign adult familial myoclonic epilepsy (10) that encodes a long multi-repeat protein (Figure 3C). *CSMD3* has been found recurrently mutated across several cancer types and, therefore, has been predicted as a cancer driver by several methods (Figure 3D). Because of its length, sequence composition and location in proximity of fragile sites of the genome, *CSMD3* was regarded as a possible false positive in NCG 4.0. The fact that *CSMD3* is constitutionally not expressed in many tissues where it is mutated (Figure 3E) also supports the passenger role of the acquired mutations. Despite this, however, the stable knockout of *CSMD3* in immortalized epithelial cells has been reported to increase cell proliferation (11), thus suggesting a tumour-suppressor role for this gene. This example highlights the difficulty to correctly predict the driver role of mutated genes and the need of multiple independent pieces of evidence to assess the role of mutations in cancer.

## ANNOTATION OF CANCER GENE PROPERTIES

To annotate the properties of cancer genes, original data on human genes, orthology, protein–protein and miRNA interactions and gene expression have been updated (Table 2).

**Table 2.** Data and properties of cancer genes in NCG 5.0

Data sets in NCG 5.0		All cancer genes (1571)	Known cancer genes (518)		Candidate cancer genes (1053)	Other human genes
			Dominant (395)	Recessive (112)		
Human genes	All genes	1525	382	112	1020	17 489
	Duplicated genes (%)	280 (18%)	76 (20%)	12 (11%)	187 (18%)	3520 (20%)
Orthology	All genes	1501	379	110	1001	16 618
	Pre-metazoan genes (%)	992 (66%)	233 (61%)	80 (72%)	672 (67%)	10 516 (63%)
Protein–protein interactions	All nodes	1332	371	110	840	13 262
	Hubs (%)	558 (42%)	213 (57%)	78 (71%)	257 (31%)	2970 (22%)
	All nodes in HT network	1177	339	108	720	11 481
	Hubs in HT network (%)	386 (33%)	148 (44%)	52 (48%)	177 (25%)	2681 (23%)
Protein complexes	Proteins (%)	752 (49%)	238 (62%)	87 (78%)	418 (41%)	4917 (28%)
miRNA interactions	miRNA target genes (%)	1101 (72%)	332 (87%)	99 (88%)	662 (65%)	10 643 (61%)
	miRNAs	324	247	163	250	438
Expression in normal tissues	All genes in GTEx	1513	379	111	1012	16 818
	Ubiquitous genes (%)	965 (64%)	301 (79%)	98 (88%)	555 (55%)	11 077 (66%)
	Tissue-specific genes (%)	62 (4%)	5 (1%)	0 (0%)	57 (6%)	726 (4%)
	All genes in Protein Atlas	1517	378	112	1016	16 889
	Ubiquitous genes (%)	831 (55%)	278 (74%)	95 (85%)	447 (44%)	9492 (56%)
	Tissue-specific genes (%)	90 (6%)	11 (3%)	1 (1%)	78 (8%)	1042 (6%)
Expression in cancer cell lines	Cancer cell line encyclopedia	1426	367	106	942	15 158
	COSMIC Cancer Lines	1398	358	105	924	14 788
	Genentech data set	1524	381	112	1020	17 164

Of the 518 known cancer genes derived from CGC, 391 are annotated as dominant (mostly oncogenes), 108 as recessive (mostly tumour-suppressors), four as both as dominant and recessive and 15 have no specified mode of action. Duplicated genes have one or more duplicated loci in the genome covering ≥60% of their length (12). Pre-metazoan genes originated in the Last Universal Common Ancestor, Eukaryotes or Opisthokonts. Ubiquitously expressed genes are expressed in ≥95% tissues (29 tissues in GTEx and 30 tissues in Protein Atlas). HT = high throughput (publications reporting ≥100 interactions).

Applying the previously described method (12), protein sequences from RefSeq v.63 (13) were aligned to the human genome assembly Hg19 to identify unique gene loci. These included 1525 of the 1571 cancer genes (13 cancer genes did not have RefSeq entries and 33 had no match in Hg19 or were gene isoforms). Cancer genes confirm their lower duplicability as compared to non-cancer genes and the signal derives from recessive cancer genes (*P*-value = 0.02, chi-square test, Table 2).

Orthology information from EggNOG v.4 (14) was used to trace the evolutionary origin of 1501 cancer genes, as described earlier (15). In line with previous reports (15–17), a higher fraction of cancer genes have orthologs in pre-metazoan species as compared to other human genes (*P*-value = 0.03, chi-square test, Table 2).

Four sources of primary interaction data (BioGRID v.3.4.125 (18); MIntAct v.190 (19); DIP (April 2015) (20); HPRD v.9 (21)) were integrated to rebuild the human protein–protein interaction network. This network included 1332 cancer proteins, which encode a higher fraction of hubs (defined as 25% most connected nodes of the network) as compared to other human proteins (*P*-value = 2.7 × 10^−56^, chi-square test, Table 2). We verified that cancer genes encode a higher fraction of protein hubs also in the network derived from high-throughput screenings (*P*-value = 7.7 × 10^−13^, chi-square test, Table 2). This excludes biases due to the higher number of single-gene experiments involving cancer proteins.

To complete the annotation of protein–protein interactions, NCG now collects also information on 752 cancer proteins involved in complexes as gathered from three resources (CORUM (February 2012) (22), HPRD v.9 (21), Reactome v.53 (23)). Supporting the signal from the overall protein–protein interaction network, a higher percentage of cancer proteins engage in complexes as compared to non-cancer proteins (*P*-value = 3.0 × 10^−67^, chi-square test, Table 2).

Interactions between 324 miRNAs and 1101 cancer genes were derived from miRTarBase v.4.5 (24) and miRecords (April 2013) (25). Similarly to the protein–protein interaction network, also in the miRNA network a significantly larger fraction of cancer genes are target of miRNAs as compared to other human genes (*P*-value = 3.0 × 10^−18^, chi-square test, Table 2).

This release of NCG provides information on the expression of cancer genes in normal tissues and in cancer cell lines. For normal tissues, NCG relies on GTEx v.1.1.8 (26) and Protein Atlas (April 2015) (27), which both derive gene expression from RNASeq data in a total of 38 tissues. Expression values (FPKM for GTEx and RPKM for Protein Atlas) were used to derive expression categories (low, medium and high expression) for each gene and to calculate the distribution of gene expression across samples in each tissue. In both data sets, larger fractions of known cancer genes, but not of candidate cancer genes, are ubiquitously expressed (expression in >95% of all tissues) as compared to other genes (*P*-value = 1.3 × 10^−13^ and *P* = 1.3 × 10^−19^ for GTEx and Protein Atlas, respectively, chi-square test, Table 2). Conversely, significantly lower fractions of known cancer genes, but not of candidate cancer genes, are tissue specific (*P*-value = 4.2 × 10^−4^ and *P*-value = 6.9 × 10^−4^, for GTEx and Protein Atlas, respectively, chi-square test, Table 2).

Three data sets (Cancer Cell Lines Encyclopedia (28), COSMIC Cancer Lines Project (29) and the recently released Genentech data set (30)) were used to derive gene expression in a total of 1543 cancer cell lines (Table 2). For each cancer gene, NCG provides the original expression value in each cell line as well as the normalized expression score, calculated as previously reported (31).

## DATA ACCESS

NCG web interface has been reorganized, with particular focus on the summary of gene information and on the visualization of gene expression profiles. The gene summary now includes additional cross-references to external resources on protein domain architecture (32), drug and compound interactions (33,34) and protein druggability (35). For each cancer gene, the type of mutational screenings, the supporting methods and any experimental validation are detailed. Gene expression profiles are now shown as interactive graphs reporting the distribution of expression levels in each normal tissue and as summary tables in cancer cell lines.

NCG website provides overview statistics of the data contained in the database, including the list of 49 cancer types and corresponding 24 primary sites, the distribution of known and candidate cancer genes per primary sites, and information on 48 possible false positives. These include 14 genes derived from the literature (6), 4 additional genes that likely accumulate a high number of alterations due to their length and 30 olfactory receptor genes. All data contained in the database can be exported in batch using the advanced search option.

## NCG USAGE

**Figure 4.**
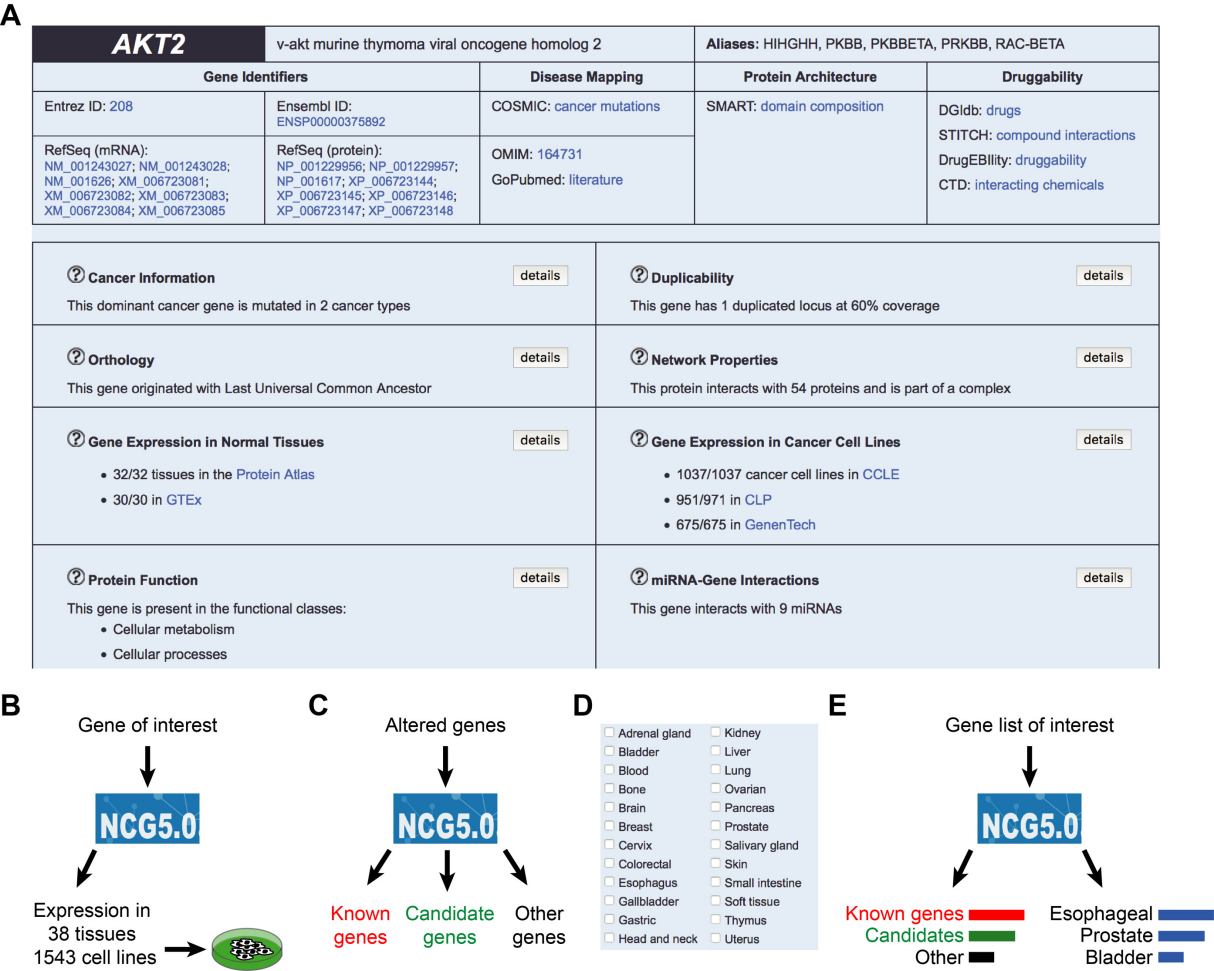
Examples of NCG usage: (**A**) Example of information available in NCG for a given cancer gene, in this case the oncogene *AKT2*. NCG summarizes the gene mutation profile across cancer types, information on duplicability, orthology, protein–protein and miRNA interactions and gene expression (**B**) NCG can facilitate the selection of the best cell systems for experimental assays by providing the expression profile of the gene of interest in several tissues and cell lines. (**C**) NCG can be used to annotate altered genes from mutational screenings. (**D**) The advanced search interface of NCG allows the identification of drivers in a variety of cancer types. (**E**) NCG can be integrated in gene enrichment analysis pipelines as a source of cancer genes.

NCG offers a multi-level annotation of cancer genes that can be queried to gain insights on mutation status, properties, function and expression profiles of cancer genes (Figure 4A). This information facilitates the characterization of cancer genes and associated features. For example, gene duplicability has been exploited to extract duplicated tumour suppressor genes and to verify the occurrence of negative epistasis between them and their paralogs (36). Another useful feature of NCG is the comprehensive overview of gene expression profiles across a vast range of normal tissues and cancer cell lines. This can guide the selection of the most adequate cell systems for planning *in vitro* experiments (Figure 4B).

NCG is exploited widely as a repository of cancer genes (17,37–50). Examples include the use of NCG to test for the proximity of cancer genes to retrovirus insertion sites (48) and to evaluate the features of cancer classification methods (41). NCG also facilitates the interpretation of cancer mutational screenings by annotating the properties of mutated genes (Figure 4C) overall and in selected cancer types (Figure 4D). For example, NCG has been used to verify whether genes undergoing copy number variations in familial breast cancer were already known cancer genes (49). Finally, NCG can be easily integrated into more complex analytical pipelines (Figure 4E). In the method developed by Zeller *et al*., NCG provides a source of true cancer genes to prioritize drivers (50). In the DOSE bioconductor package, NCG is implemented as a source of cancer genes to perform enrichment analysis (51).

## FUTURE WORK

It is expected that mutational screenings of cancer samples will continue to produce large amounts of data in the next years. The launch of personal genome initiatives ((52) and www.genomicsengland.co.uk) and the delivery of pan-cancer projects will substantially enlarge the spectrum of cancer types and samples with available mutational profiles. This will allow the discovery of novel cancer genes, particularly of those that recur in few samples and are currently difficult to identify. In parallel, the development of novel approaches for high-throughput functional screenings (e.g. based on the CRISPR-Cas technology (53–56)) promises to improve the efficiency of experimental validation assays.

In this exciting scenario, NCG will continue in its commitment to manually curate the literature to extract cancer genes and annotate available orthogonal supports. NCG will also expand to include other types of cancer driver alterations, such as copy number variations, gene rearrangements and non-coding modifications (57,58). In addition to enlarge the repertoire of cancer drivers, NCG will integrate new properties, e.g. the epigenetic regulation of cancer genes and their germline mutations.

As data become available, NCG will include the clinical relevance of cancer genes, such as their actionability as pharmacological targets (59) and their applicability as biomarkers of cancer progression. All these efforts will contribute towards a more complete characterization of the molecular determinants of cancer.
